# Can a Simple Dietary Index Derived from a Sub-Set of Questionnaire Items Assess Diet Quality in a Sample of Australian Adults?

**DOI:** 10.3390/nu10040486

**Published:** 2018-04-13

**Authors:** Alexia Bivoltsis, Georgina S. A. Trapp, Matthew Knuiman, Paula Hooper, Gina L. Ambrosini

**Affiliations:** 1 School of Population and Global Health, The University of Western Australia, 35 Stirling Hwy, Crawley, WA 6009, Australia; gina.trapp@telethonkids.org.au (G.S.A.T.); matthew.knuiman@uwa.edu.au (M.K.); gina.ambrosini@uwa.edu.au (G.L.A.); 2 Telethon Kids Institute, The University of Western Australia, PO Box 855, West Perth, WA 6872, Australia; 3 School of Agriculture and Environment and the School of Human Sciences, The University of Western Australia, 35 Stirling Hwy, Crawley, WA 6009, Australia; paula.hooper@uwa.edu.au

**Keywords:** diet quality, diet quality index, dietary methods, Australian

## Abstract

Large, longitudinal surveys often lack consistent dietary data, limiting the use of existing tools and methods that are available to measure diet quality. This study describes a method that was used to develop a simple index for ranking individuals according to their diet quality in a longitudinal study. The RESIDential Environments (RESIDE) project (2004–2011) collected dietary data in varying detail, across four time points. The most detailed dietary data were collected using a 24-item questionnaire at the final time point (*n* = 555; age ≥ 25 years). At preceding time points, sub-sets of the 24 items were collected. A RESIDE dietary guideline index (RDGI) that was based on the 24-items was developed to assess diet quality in relation to the Australian Dietary Guidelines. The RDGI scores were regressed on the longitudinal sub-sets of six and nine questionnaire items at T4, from which two simple index scores (S-RDGI1 and S-RDGI2) were predicted. The S-RDGI1 and S-RDGI2 showed reasonable agreement with the RDGI (Spearman’s rho = 0.78 and 0.84; gross misclassification = 1.8%; correct classification = 64.9% and 69.7%; and, Cohen’s weighted kappa = 0.58 and 0.64, respectively). For all of the indices, higher diet quality was associated with being female, undertaking moderate to high amounts of physical activity, not smoking, and self-reported health. The S-RDGI1 and S-RDGI2 explained 62% and 73% of the variation in RDGI scores, demonstrating that a large proportion of the variability in diet quality scores can be captured using a relatively small sub-set of questionnaire items. The methods described in this study can be applied elsewhere, in situations where limited dietary data are available, to generate a sample-specific score for ranking individuals according to diet quality.

## 1. Introduction

Diet is a major modifiable risk factor for a range of chronic diseases [1]. Analysis of diet in large populations is crucial for identifying dietary aspects that can increase or decrease the risk of chronic disease. Studies are recognizing the importance of characterizing the total diet, rather than single nutrients or foods, since a wide range of dietary components play a role in the development of chronic diseases, such as obesity, cardiovascular disease, diabetes, and some cancers [2,3,4]. As such, within nutritional epidemiology, there is a trend towards the use of single indices that provide an overall assessment of diet quality [5]. Diet quality indices describe the nutritional adequacy of an individual’s diet as it adheres to pre-defined national dietary guidelines [6,7]. A measure of diet quality that is based on national dietary guidelines can be used as a predictor of chronic disease, in order to investigate associations with other behaviors, as a control in epidemiological research, or to examine diet-exposure relationships.

Detailed methods, such as multi-item, semi-quantitative food frequency questionnaires (FFQ), three-day food records, and 24-h recalls are widely used for the assessment of diet quality [8]. Whilst these tools provide information on food and nutrient intake, they are often time consuming and too resource intensive for use in large epidemiological research settings. Therefore, short dietary questionnaires are increasingly used to assess diet quality in large population surveys. Rather than quantifying actual intakes, short dietary questionnaires aim to rank individuals according to their dietary intake. A number of short dietary questionnaires or screeners have been successfully developed and validated, demonstrating the utility of such tools for the assessment of certain food groups [9,10,11,12,13], nutrients [10,14,15,16], specific dietary patterns [17,18], or diet quality [19,20,21,22,23,24,25]. Furthermore, several approaches have been taken to reduce the length of existing dietary questionnaires whilst still maintaining the validity of reduced-item scores. These involve regression methods [26,27,28], data reduction techniques, such as factor analysis [29,30] and principal component analysis [31], or the use of correlations between nutrient estimates from full and reduced versions [32].

However, in large scale, longitudinal studies, inconsistent dietary data are often collected at successive time points. This presents a challenge when attempting to make longitudinal assessments of diet quality, owing to lack of consistent reporting. Thus, this study investigates whether it is possible to construct a longitudinal measure of diet quality in situations where inconsistent dietary data are available across time points.

In the RESIDential Environments (RESIDE) project, which is an Australian longitudinal study of adult health and behavior, dietary data were collected at four time points in varying detail. The most comprehensive dietary data were obtained at the fourth and final time point (T4) in the form of a 24-item questionnaire containing questions about dietary behaviors and the frequency of consumption of selected foods and food groups. Preceding time points collected dietary data on sub-sets of the 24 items. Given that full dietary data were not available across all of the time points, a method of regression was used to construct a longitudinal measure of diet quality from the sub-sets of dietary data. The aim was to demonstrate that a simple diet quality index based on a sub-set of the 24 RESIDE questionnaire items, can rank individuals according to their diet quality, as determined by a more comprehensive index that was designed to reflect adherence to the Australian Dietary Guidelines.

## 2. Materials and Methods

### 2.1. Study Design and Participants

This study used data from the RESIDential Environments (RESIDE) project. RESIDE was a quasi-experimental, longitudinal study that was conducted across Perth, Western Australia (WA), from 2003 to 2012. The primary aim of RESIDE was to evaluate the impact of the Western Australian government’s new sub-division design code policy “Livable Neighborhoods Community Design Guidelines” (LNG) on participant health and behavior. A cohort of adults (*n* = 1811) moving from their house located within an established residential area into one of 74 new housing developments were surveyed at four times; T1 prior to moving (baseline from 2003–2005), T2 (1-year post move from 2004–2006), T3 (2–3 years post from 2006–2008), and T4 (6–9 years post move from 2011–2012). At each time point (T1, T2, T3 and T4), the participants completed questionnaires on self-reported physical activity, health, lifestyle behaviors, usual food intake, and sociodemographic variables. Of the 1811 participants at baseline (40% male), ages ranged from 19–78 years with a mean age of 41 (SD = 13) in men and 39 (SD = 11) in women. Detailed descriptions of the study design and sampling procedures are presented elsewhere [33]. The current study utilized cross-sectional data (*n* = 565) from T4. Ethics approval was provided by the University of Western Australia Human Research Ethics Committee.

### 2.2. Sociodemographic, Health and Lifestyle Variables

Self-reported sociodemographic data at T4 included gender, age (years), marital status (married/de facto; separated/divorced/widowed; single), education (secondary or less; trade/apprentice/certificate; bachelor or higher), and income (<50,000; 50,000–69,999; 70,000–89,999; ≥90,000). Health and lifestyle variables included smoking status (participants were asked, “Which of the following best describes your cigarette smoking status: (1) I smoke daily; (2) I smoke occasionally = current smoker; (3) I don’t smoke now but I used to; (4) I’ve tried it a few times but never smoked regularly = ex-smoker; and, (5) I’ve never smoked = never smoked), self-rated health (participants were asked “In general, would you say that your health is excellent, very good, good, fair, or poor?”), and body mass index (BMI) based on self-reported height and weight categorized into healthy: BMI ≥ 18.5 to <25 kg/m^2^; overweight: BMI ≥ 25 to <30 kg/m^2^; and, obese: BMI ≥ 30 kg/m^2^ [34]. The frequency and intensity of physical activity was also assessed in minutes per week. Participants reported the number of times and minutes per week of either walking (for recreation and transport), and moderate or vigorous intensity leisure time activities. These data were then used to derive an overall measure of physical activity using standardized scoring and levels of activity [35,36].

### 2.3. Dietary Intake

At T4, the participants completed a 24 item dietary assessment that included 12 semi-quantitative food frequency questions (FFQ) and 12 dietary behavior questions (DBQ). At previous time points (T1, T2 and T3) data were collected on a sub-set of the T4 survey items (Table A1 in Appendix A). All of the dietary survey items were sourced from questions from the 1995 Australian National Nutrition Survey [37,38] or previously evaluated questions that were shown to be valid measures of food intake and dietary behaviors [39,40,41,42]. A one-week test-retest of survey items provided intraclass correlations (ICC) ranging from 0.79 to 0.95 [43]. The standard serving size of fruit, vegetables, and beverages was defined in the surveys in terms of metric measurements (cups and milliliters), and included additional information to guide the participants about how many cups in everyday drinks, i.e., 1 can of soft drink = 1.5 cups, 1 bottle of Gatorade = 2 cups, and 1 bottle of soft drink = 2.5 cups.

### 2.4. Assessment of Diet Quality

Diet quality was assessed at T4 using three indices: a RESIDE dietary guideline index (RDGI), developed using the most comprehensive data (24-items) available at T4; and two simple RESIDE dietary guideline indices (S-RDGI1 and S-RDGI2) constructed using the sub-sets of six survey items available at T1, T2, T3, and T4 (S-RDGI1), and nine survey items available at T2, T3, and T4 (S-RDGI2).

Development of the RDGI score: The RDGI was adapted from previously validated food-based indices that were developed to reflect the Australian Dietary Guidelines (ADG) [44,45,46] and was designed to assess the adherence with components of the ADG relevant to adult (>18 years) dietary intake [4], and the Australian Guidelines to Reduce Health Risks from Drinking Alcohol [47]. All 24 survey items contributed to the RDGI, and were assigned as indicators for each ADG component. A total of 10 ADG components contributed to the RDGI score, including six adequacy components (i.e., foods to increase in the diet as per ADG 2: Enjoy a wide variety of nutritious foods from these five groups every day) and four moderation components (i.e., foods to limit in the diet as per ADG 3: Limit intake of foods containing saturated fat, added salt, added sugars, and alcohol) (Table 1). See Table A2 for relevant guidelines and components used to construct the RDGI.

The aim of scoring and cut-off points was to achieve a balanced contribution of components to the overall score that resulted in high discriminating power. Using data-driven group or population medians as cut-offs may provide greater discriminating power, but are not comparable across groups and may not be related to recommended intakes as per guidelines. This study aimed to assess diet quality in relation to the ADG. Thus, cut-offs reflected age and sex-specific ADG recommendations for number of serves. Each component was scored from 0–10, such that equal weights were attributed to all of the components within the index. This is the common approach for most dietary indices [6].

When components consisted of more than one indicator item, scores totaled to a maximum of 10. The maximum score was assigned when the guideline was met, with a proportionate score given for levels of intake above/below this, and zero assigned as the minimum score (furthest from guideline). Items were scored proportionately, to the extent to which the guideline was met, to allow for the final score to reflect the degree to which individuals met recommendations. For example, for fruits and vegetables, maximum points (*n* = 10) were assigned to intakes at or above recommendations (number of serves per day), and a minimum score of zero for non-consumers. This is consistent with evidence that suggests an inverse dose-response association between intake of fruits and vegetables with the risk of coronary heart disease, stroke, cardiovascular disease, total cancer, and all-cause mortality [48]. For components that were shown to follow a U-shaped association with health, i.e., red meat [49], maximum points were scored to participants at or below the recommended intakes, and a lower proportionate score was given to intakes greater than recommendations. This was done so as not to penalize individuals who chose not to consume alcohol or red meat for various reasons. In situations where the ADG were not quantitative, or did not align with the response categories of the survey items, criteria provided from another set of national recommendations or empirical study were sought to inform cut-offs. Cut-offs for maximum and minimum scores are detailed in Table 1.

This scoring approach was similar to that of McNaughton et al. (2008) and Thorpe et al. (2016), and recommended dietary index methodology, which recognizes the existence of correlations and interactions between individual dietary components such that strongly correlated dietary variables contribute more heavily to the score [6,44,46]. The final total score was the sum of the 10 components so that the index ranged from 0–100, with a higher score reflecting greater diet quality in relation to the ADG.

Development of the S-RDGI1 and S-RDGI2 scores: The sub-sets of six survey items available at T1, T2, T3, and T4 are highlighted in Table 1 and the additional three items available at T2, T3, and T4 are in bold italic (nine survey items in total available at T2, T3, and T4). The six survey items totaled to a maximum score of 31.5, whilst the nine survey items totaled to a maximum score of 46.5. In order to generate a representative measure of diet quality (RDGI) for use across all of the time points, a method of linear regression was used to examine the relationship between the available sub-sets of scores and the full RDGI, from which the fitted regression model can be used to predict the dependent variables (S-RDGI1 and S-RDGI2) at time points when only the independent variables (sub-sets of scores) are known. A multiple linear regression model was fitted using the RDGI scores (dependent variable) and the scores of the six survey items (independent variables) at T1, T2, T3, and T4 (Table 1). The resultant S-RDGI1 score was calculated as the predicted index score from the estimated regression equation. The same procedure was repeated to determine the S-RDGI2 score using the nine survey items that are available at T2, T3, and T4 (Table 1).

### 2.5. Statistical Analysis

From the 565 participants at T4, 555 (212 men and 343 women) provided complete responses to all of the 24 dietary survey items that were required to calculate the RDGI scores and undertake subsequent analyses.

Descriptive statistics were calculated for participant sociodemographic, health and lifestyle variables, RDGI scores, and ADG components, along with the percentage of participants that met maximum criteria for each ADG component. Using the outputs that were obtained from the fitted linear regression models, the respective S-RDGI1 and S-RDGI2 scores were calculated using the model intercept plus the sum of the corresponding survey item scores multiplied by their regression coefficient, e.g., Y’ = βX + A. Where Y’ equals the predicted index score (S-RDGI1 or S-RDGI2), β is the corresponding regression coefficient for the independent variable X (survey item score) and A is the intercept.

Several approaches were used to assess the agreement between RDGI scores and S-RDGI scores (S-RDGI1 and S-RDGI2) [53]. Spearman’s rank correlation coefficients were used to assess the strength of the associations between diet scores (RDGI, S-RDGI1, and S-RDGI2) and the intakes of food and drink items and ADG component scores. The following categories were used for the interpretation of correlation coefficients: 0.9–1 almost perfect; 0.7–0.9 very high; 0.5–0.7 high; 0.3–0.5 moderate; 0.1–0.3 low; and, 0–0.1 insubstantial [54].

Cross-classification was used to determine the percentage of participants that were correctly classified by the S-RDGI1 and S-RDGI2 into the same tertile (good outcome = ≥ 50%) or grossly misclassified into the opposite tertile (good outcome = ≤ 10%) of diet quality, as measured by the RDGI. Since this method of cross-classification may include agreement that has occurred by chance, Cohen’s weighted kappa (Kw) was also calculated to provide a measure of agreement that is adjusted for chance where kw = (Po − Pe/(1 − Pe)), Po = observed agreement, and Pe = expected agreement by chance. Weights were applied such that no difference between tertiles = 1, a difference of one tertile = 0.5 and a difference of two tertiles = 0. Values of Kw > 0.8 indicate very good agreement, between 0.8–0.61 good agreement, 0.6–0.41 moderate agreement, 0.40–0.21 fair agreement, and <0.2 poor agreement [54].

Bland-Altman plots further examined the agreement between S-RDGI scores (S-RDGI1 and S-RDGI2) and RDGI scores [55]. The difference between the scores (S-RDGI − RDGI) was plotted against the mean of both the scores ((S-RDGI + RDGI)/2), for both S-RDGI1 and S-RDGI2. The mean difference and 95% limits of agreement (LOA) were calculated (±1.96 SD) to examine if the mean difference varied with diet score. Linear regression analysis of the difference on the average of the two measures examined whether the mean difference varied significantly with diet score.

Associations between diet scores (RDGI, S-RDGI1, and S-RDGI2) and participant characteristics (gender, age, education, income, self-rated health, smoking status, physical activity, and weight status) were tested using analysis of variance (ANOVA), with *post hoc t*-tests if more than two categories. All of the analyses were conducted using IBM SPSS Statistics for Windows, Version 23.0. Armonk, NY: IBM Corp.

## 3. Results

### 3.1. Sample Characteristics

The distributions of participant characteristics are shown in Table 2. The mean age was 48 years (range = 25–80). Greater than 85% were married or in a de facto relationship, and over half had greater than secondary school education or an income ≥ $90,000. More than 50% of participants rated their health as very good or excellent, only 6.8% were current smokers, and over 55% of participants were classified as either overweight or obese. The mean total amount of moderate to vigorous physical activity per week was 5.6 h. The mean RDGI score was 69.9 (SD = 8.8) and the distribution was slightly negatively skewed (skewness = −0.33). Participants scored the highest on the saturated fat, water/fluids, alcohol, and fruit components. Overall, there were few ADG components that were well met by participants, with fruit (53.5%) and alcohol (46.7%) having the greatest percentage of participants achieving maximum scores.

### 3.2. Development of the S-RDGI1 and S-RDGI2 Scores

Table 3 shows the estimated coefficients from the fitted linear regression models for the prediction of S-RDGI scores from the scores of the six survey items (S-RDGI1) and nine survey items (S-RDGI2). All six and nine items had a coefficient that was significantly different from zero (*p* < 0.05). All of the coefficients were positive, indicating that a higher diet quality was associated with higher intakes of vegetables, fruit, fish and reduced fat dairy and lower intakes of red meat, chips, meat products, and alcohol. For both of the models, fruit then vegetables had the greatest effect on diet quality scores (based on standardized coefficients, i.e., coefficient *SD = standardized coefficient), followed by alcohol (days/week), red meat and milk type for the S-RDGI2, and red meat then milk type for the S-RDGI1. Overall, 62% of the variation in the RDGI scores in this sample was explained by the six survey items (R^2^ = 0.62, *p* < 0.001) and 73% was explained by the nine survey items (R^2^ = 0.73, *p* < 0.001). The mean diet quality score was 69.8 (SD = 8.8) for the RDGI, 69.8 (SD = 6.9) for the S-RDGI1, and 69.8 (SD = 7.5) for the S-RDGI2.

### 3.3. Agreement between RDGI and S-RDGI Scores

Correlations between the diet quality scores (S-RDGI1, S-RDGI2 and RDGI) and specific food and drink items and ADG component scores are presented in Table 4. Intakes of fruit and vegetables were highly positively correlated with all of the diet quality scores. Whereas, the intakes of discretionary foods, including: chips; fried roast or BBQ chicken, pizza, burgers, or fish and chips; and, meat pies, sausage rolls, or other savory pastries, were highly, negatively correlated with all of the diet scores. As expected, food and drink items that were not included in the S-RDGI1 and S-RDGI2 were not strongly correlated with these scores.

The S-RDGI1 scores correctly classified 360 participants (64.9%) into the same tertile, misclassified 185 (33.3%) into adjacent tertiles, and grossly misclassified 10 (1.8%) into opposite tertiles of the RDGI. The S-RDGI2 scores correctly classified 387 participants (69.7%) into the same tertile, misclassified 158 (28.4%) into adjacent tertiles, and grossly misclassified 10 (1.8%) into opposite tertiles of the RDGI. The weighted k-statistic showed that the RDGI and S-RDGI1 had moderate agreement (Kw = 0.58; 95% CI = 0.53, 0.64; *p* < 0.001), and the RDGI and S-RDGI2 had good agreement (Kw = 0.64; 95% CI = 0.59, 0.69; *p* < 0.001).

**Figure 1:**
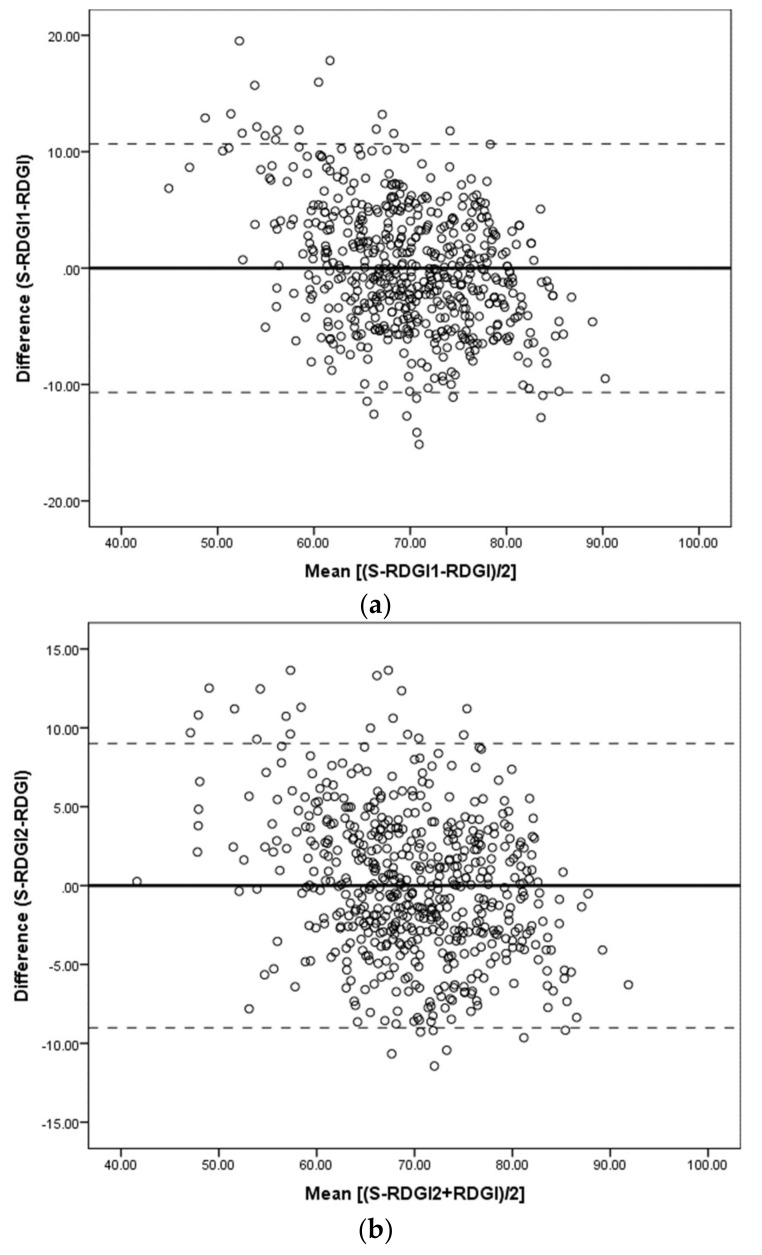
Bland-Altman plot showing agreement between diet quality scores calculated by the RDGI and: (**a**) S-RDGI1; (**b**) S-RDGI2. – Mean difference, – – – 95% limits of agreement (LOA).

The relationship between the difference in diet scores and their mean (Bland-Altman plot) is illustrated in Figure 1a (S-RDGI1 and RDGI) and Figure 1b (S-RDGI2 and RDGI). The S-RDGI1 and S-RDGI2 appear to over-estimate the lower RDGI scores and under-estimate the higher RDGI scores. The slope of the fitted regression line for the difference versus the means of diet scores was significantly different from zero for both the S-RDGI1 (slope β = −0.269; *p* < 0.001) and S-RDGI2 (slope β = −0.171; *p* < 0.001).

### 3.4. Associations between Diet Scores and Participant Characteristics

Mean diet quality scores are shown in Table 5 for the RDGI, S-RDGI1, and S-RDGI2, according to categories of sociodemographic, health, and lifestyle characteristics. A higher diet quality, as determined by the RDGI, S-RDGI1, and S-RDGI2, was associated with being female, undertaking ≥150 min of physical activity per week, and being in excellent health (compared with good/fair). Current smokers had significantly lower diet quality when compared with ex-smokers or those who had never smoked. Those who were overweight or obese had lower mean diet quality, however, this was not statistically significant. Similarly, although there was an increasing trend in mean diet quality for both RDGI and S-RDGI1 with an increasing education level, this was not significant. Diet quality tended to increase with age. There was no significant association or trend in mean diet quality between RDGI, S-RDGI1, and S-RDGI2 and income.

## 4. Discussion

Using a method of regression, we found that diet quality scores that were calculated using a sub-set of six (S-RDGI1) and nine (S-RDGI2) survey items showed good agreement with those that were derived from the full 24-item questionnaire (RDGI). All three indices (S-RDGI1, S-RDGI2, and RDGI) showed expected variations across sociodemographic, health, and lifestyle variables (with the exception of income and education), and correlated well with intakes of key food and drink items and ADG component scores. Overall, 62% of the variation in the RDGI scores was explained by the six survey items that were included in the S-RDGI1 and 73% was explained by the nine survey items that were included in the S-RDGI2. Gross misclassification was very low, correct classification was high, and kappa scores were moderate to good.

Diet scores from all three indices (S-RDGI1, S-RDGI2, and RDGI) were significantly correlated with most of the ADG components and the intakes of key food and drink items that are consistent with a healthy diet (i.e., as diet scores increased, intakes of fruit, vegetables, and fish increased, whereas the intakes of red meat, discretionary foods, and alcohol decreased). Correlations ranged from very high to moderate, with the exception of biscuits, cakes, desserts, pastries, lollies, and/or chocolate and sugary drinks. Although not directly comparable, due to variations in methodology, the correlation coefficients from this study were in range with those that were reported elsewhere in the literature [56,57,58,59].

Food and drink items that were not included in the S-RDGI1 and S-RDGI2 were not strongly correlated with these scores, neither were underrepresented ADG components (i.e., grains/cereals). This was expected given that the simple index scores included only a sub-set of survey items and they were not designed to adequately measure intakes of food groups but to differentiate individuals based on their diet quality. Intakes of bread; pasta, rice, noodles, or other cooked cereals; milk; and, cheese did not correlate well with all three indices. Furthermore, the corresponding ADG components for grains/cereals and dairy or alternatives did not correlate well with all scores. It is possible that insufficient detail (e.g., portion sizes, reduced fat versus full fat, and whole grains versus refined grains) being collected on these dietary components limited their contribution to discriminating individuals with high or low diet quality. Alternatively, these dietary components may not be important indicators of diet quality. Other studies have reported similar findings with intakes of grains and dairy showing weak correlations with diet quality [56,59,60].

Both the S-RDGI1 and S-RDGI2 over-estimated lower RDGI scores and under-estimated higher RDGI scores. This bias was less apparent for the S-RDGI2, as expected given the inclusion of three extra survey items in the S-RDGI2. However, achieving absolute agreement between the scores was not the main aim of this study, rather, it was intended to rank participants according to their diet quality. Results showed that 95% of participants had a difference in diet quality scores within ±11 (S-RDGI1) and ±9 (S-RDGI2) points of the RDGI, and this is adequate to successfully classify individuals into high, medium, or low diet quality.

There were few ADG components that were well met by participants, as consistent with previously published Australian work [44,46,61]. Participants scored higher on components for alcohol and fruit intake, and lower for vegetables, grains/cereals, and dairy components. Other studies have reported similar findings [44,46], as did the 2014 Health and Wellbeing Survey for adults in Western Australia, in which 52% and 8.8% of respondents reported eating the recommended daily serves of fruit and vegetables, respectively [62].

Both the S-RDGI1 and S-RDGI2 performed comparatively well in distinguishing between groups with known differences in diet quality. For all of the indices, a higher diet quality was associated with being female, undertaking moderate and high amounts of physical activity, not smoking, and being in excellent health. These findings are consistent with previous studies [44,46]. The lack of a significant association between diet quality indices and either income or education was likely due to the characteristics of the RESIDE study sample being of a relatively higher socioeconomic status with over half having greater than secondary school education or an income ≥ $90,000. Similarly, the lack of a clear trend in diet quality with age for all of the indices may be due to the disproportionate sample, with only 12 participants being under the age of 30. However, diet quality was significantly higher in the oldest age group for the S-RDGI1 and S-RDGI2, as seen in the latest 2011–2012 National Nutrition and Physical Activity Survey (NNPAS) [63]. Although participants that were classified as overweight or obese had lower diet quality, as determined by all three indices, this was not significant and was possibly due to the unreliable nature of self-reported height and weight data [64], or dietary under-reporting being more common in overweight people [65].

### 4.1. Implications

The S-RDGI2 (nine item score) performed slightly better than the S-RDGI1 (six item score) across all of the measures of agreement. However, the addition of three extra survey items in the S-RDGI2 for fish and alcohol intake only improved the regression model R^2^ by 11%. Since the S-RDGI1 demonstrated good agreement with similar results to the S-RDGI2, this suggests that a large proportion of the variability in diet quality can be explained by relatively few survey items relating to dietary behaviors, consumption, and food frequency. These findings are consistent with previous studies. For example, the UK Eating Choices Index (ECI), which developed from a four-item questionnaire, successfully discriminated between healthy and unhealthy eating choices and was significantly correlated with nutrient profiles consistent with a healthy diet [25]. Similarly, an eight-item Danish dietary quality score (DQS) was associated with key nutrients and biomarkers that are consistent with higher diet quality [66]. Indeed, research has shown that intakes of certain food types can significantly predict an individual’s diet quality [24]. This has implications for the development of new short dietary questionnaires or screeners, suggesting that just a few key questions relating to individual food items can be successfully used to classify populations or groups according to their diet quality.

### 4.2. Strengths and Limitations

This study was limited by the modest response rate (31%), and participant characteristics may not be comparable to national statistics due to the nature of the RESIDE study design. As such, results may not be transferable to other populations. Furthermore, it is acknowledged that given the low response rate and the period of data collection that was used in this study, differences in food consumption patterns amongst groups of people (responders and non-responders) or over time, may mean that the six and nine-item questionnaires do not predict diet quality as effectively in situations outside the present study. Rather, researchers should apply the techniques that are described in this paper to an existing dataset in order to generate a sample-specific measure of diet quality. No information on portion sizes of food frequency items was obtained, nor was it possible to adjust scores by energy intake. However, this is consistent with brief dietary assessment methods and other studies [44,46]. The present study did not measure dietary variety across food groups. Studies have demonstrated variety across food groups to be a stronger predictor of dietary quality than variety within those groups [67]. However, the inclusion of dietary variety at the food group level can be considered to be unnecessary since only with a varied diet is it possible to score high on all food group items [6]. In addition, individuals with high intakes as a result of higher energy needs will more likely also have a higher dietary variety. There was limited dietary information on unsaturated fats and meat alternatives, e.g., nuts, seeds, beans/legumes, tofu, or eggs. Therefore, these aspects of diet were underrepresented in scores and may have led to the misclassification of diet quality for some participants. Similarly, fruit juice was not included as a serving of fruit on the RESIDE questionnaire and this may have resulted in the underreporting of fruit intake and contributed towards lowered scores for diet quality.

The same tool was used to create the RDGI and S-RDGI1/S-RDGI2, which can potentially lead to an overestimation of relative validity [68] and contribute to correlated errors. As such, actual agreement may be lower than the reported findings. Ideally, estimated index scores should be compared against a standard reference that is constructed from an independent dietary assessment method to avoid correlated errors [69]. However, other studies have applied similar approaches within the literature in the absence of more appropriate data [58].

This study was strengthened by the use of a food-based index of diet quality (RDGI), as consistent with the current focus of diet research [5]. Furthermore, although the RDGI was not independently validated, it was modelled on an index that has been previously validated and shown to reflect intakes of key nutrients [44]. In addition, this study used age and sex-specific cut-offs with proportionate intermediate scoring within the guidelines to incorporated additional variation in scores.

### 4.3. Recommendations for Future Research

Future research to test the reliability and validate the S-RDGI1 and S-RDGI2 against a reference method (three-day food diary and/or validated FFQ), and examine scores in relation to objectively measured health outcomes, nutrient intakes, and/or relevant biomarkers, is recommended. In addition, investigating whether the S-RDGI1 and S-RDGI2 can detect changes in diet quality over time will further evaluate the effectiveness of the described methods as a means of generating a measure of diet quality for use in epidemiological research and studies of public health interventions.

## 5. Conclusions

The S-RDGI1 and S-RDGI2 successfully ranked participants according to their diet quality and performed well across a range of measures of agreement. However, due to the discussed limitations and the potential for correlated errors, results should be interpreted with caution and we emphasize that our observations would benefit from further validation. Yet, findings indicate that a large proportion of the variability in diet quality scores can be captured using a relatively small sub-set of dietary survey items or indictor questions based on individual food groups or items. Therefore, in large-scale, longitudinal studies with limited time and resources available for dietary assessment, a sub-set of questionnaire items may be sufficient for the development of a sample-specific measure of diet quality for ranking individuals or as a confounding variable in subsequent analyses. Furthermore, in situations where incomplete dietary data are available across time points, a method of regression that is based on an available sub-set of questionnaire items may be an effective approach for generating a consistent measure of diet quality and overcoming this common limitation of longitudinal studies.

## Figures and Tables

**Table 1:** Index components and scoring criteria for the RESIDential Environments dietary guideline index (RDGI). The six survey items available at T1, T2, T3, and T4 are highlighted and the additional three items available at T2, T3, and T4 are in ***bold italic***.

ADG Component ^1^	RESIDE Indicator Survey Item	Criteria for Minimum Score	Criteria for Intermediate Score	Criteria for Maximum Score
Vegetables	How many serves of vegetables do you usually eat each day (including fresh, frozen and tinned)?	Do not eat = 0	19–70 y M: 1 serve or less = 2, 2 serves = 4, 3–4 serves = 6, 5 serves = 8 F and >70 y M: 1 serve or less = 2.5, 2 serves = 5, 3–4 serves = 7.5	19–70 y M: 6 serves = 10 F and > 70 y M: ≥ 5 serves = 10
Fruit	How many serves of fruit do you usually eat each day (including fresh, dried, frozen and tinned fruit)?	Do not eat = 0	1 serve or less = 5	≥2 serves = 10
Grains/cereals: mostly wholegrain and/or high cereal fibre varieties	What type of bread do you usually eat?	White bread = 0	Don’t eat bread/other = 1.25	High fibre white, wholemeal, multigrain, rye, spelt = 2.5
	How often do you eat bread (including bread rolls, flat breads, crumpets, bagels, English or bread type muffins)?	<once per month = 0	Once per month = 0.5 2–3 times per month = 1 1–2 times per week = 1.5 3–5 times per week = 2	6–7 times per week = 2.5
	How often do you eat pasta, rice, noodles or other cooked cereals?	<once per month = 0	Once per month = 1 2–3 times per month = 2 1–2 times per week = 3 3–5 times per week = 4	6–7 times per week = 5
Lean meats	How often do you eat red meat (beef, lamb, and kidney but not pork or ham)? Include all minimally processed forms of red meat such as chops, steaks, roasts, rissoles, mince, stir-fries, and casseroles ^2^.	6–7 times per week = 0	3–5 times per week = 2.5	≤1–2 times per week = 5 Don’t eat meat = 5
	***How often do you eat fish?*** ^3^	<once per month = 0	Once per month = 1.25 2–3 times per month = 2.5 1–2 times per week = 3.75	≥3–5 times per week = 5
Dairy or alternatives: mostly reduced fat	About how much milk (in total) do you usually have in a day?	<150 mL = 0	>70 y, 51–70 F: 150–600 mL = 1.25 19–50 y, 51–70 M: 150–300 mL = 1.25	>70 y, 51–70 F: > 600 mL = 2.5 19–50 y, 51–70 M: ≥ 301 mL = 2.5
	What type of milk do you usually consume?	If whole (full cream) = 0	If low or reduced fat/other = 1.25	If skim = 2.5
	How often do you eat cheese? (including ricotta, cottage, processed, cream cheese, hard and soft cheese)	<once per month = 0	Once per month = 1 2–3 times per month = 2 1–2 times per week = 3 3–5 times per week = 4	6–7 times per week = 5
Drink plenty of water	How many cups of water, including sparkling water, do you drink in a day?	Total beverage intake zero cups = 0	Total beverage intake: M 1–9 cups = 2.5 F 1–7 cups = 2.5	Total beverage intake: M ≥ 10 cups = 5 F ≥ 8 cups = 5
	How many cups of diet or sugar-free soft drinks, cordial or sports drinks do you drink in a day? (such as coke zero or sugar free Gatorade)			
	How many cups of hot drinks do you drink in a day? (such as tea, coffee, herbal tea)			
	Proportion of water to total beverage intake ^4^	0% = 0	>0% < 50% = 2.5	≥50% = 5
Limit intake of foods high in saturated fat	How often do you eat chips, French fries, wedges, fried potatoes or crisps?	6–7 times per week = 0	3–5 times per week = 0.5 1–2 times per week = 1 2–3 times per month = 1.5	≤once per month = 2
	How often do you eat meat products such as sausages, frankfurters, polony, meat pies, bacon or ham? ^5^	6–7 times per week = 0	3–5 times per week = 0.5 1–2 times per week = 1 2–3 times per month = 1.5	≤once per month = 2
	How often is the meat you eat trimmed of fat either before or after cooking?	Never or rarely = 0	Sometimes = 1	Usually = 2
	How often do you eat fried, roast or BBQ chicken, pizza, burgers or fish and chips? ^5^	6–7 times per week = 0	3–5 times per week = 0.5 1–2 times per week = 1 2–3 times per month = 1.5	≤once per month = 2
	How often do you eat meat pies, sausage rolls or other savoury pastries? ^5^	6–7 times per week = 0	3–5 times per week = 0.5 1–2 times per week = 1 2–3 times per month = 1.5	≤once per month = 2
Limit intake of foods and drinks containing added salt	How often do you add salt to your food after it is cooked?	Usually = 0	Sometimes = 2.5	Never or rarely = 5
	How often is salt added to your food during cooking?	Usually = 0	Sometimes = 2.5	Never or rarely = 5
Limit intake of foods and drinks containing added sugars	How often do you eat biscuits, cakes, desserts, pastries, lollies and/or chocolate ^5^	6–7 times per week = 0	3–5 times per week = 1.25 1–2 times per week = 2.5 2–3 times per month = 3.75	≤once per month = 5
	How many cups of regular or sugar sweetened soft drinks, cordial, fruit juice or sports drinks do you drink in a day? ^6^	>2 cups = 0	1.5–2 cups = 2.5	≤1 cup = 5 No response = 5
If you choose to drink alcohol, limit intake	***On how many days of the week do you usually drink alcohol?***	≥6 days per week = 0	5–2 days per week = 2.5	≤once per week = 5 Don’t drink alcohol = 5
	***On a day when you drink alcohol, how many standard drinks do you usually have?***	>4 drinks = 0	3–4 drinks = 2.5	≤2 drinks = 5

^1^ Guidelines derived from the Australian Dietary Guidelines [4]; ^2^ The Australian Dietary Guidelines recommend a maximum of 455 g (7 serves) of lean, cooked, red meat per week. However, since many Australian adults eat meat in larger portion sizes than standard serves, consuming red meat 6–7 times per week was considered greater than recommended guidelines; ^3^ The Australian Heart Foundation recommends eating fish at least 2–3 times per week [50]; ^4^ The Australian Dietary Guidelines do not provide recommendations for daily servings of beverages. Nutrient Reference Values for Australia and New Zealand were used as a guide for cut-offs. Total beverage intake excludes alcohol and sugar sweetened drinks. The proportion of water to total beverage intake was based on the methods of Thorpe et al. (2016) and McNaughton et al. (2008) derived from US beverage guidelines [44,46,51]; ^5^ The Australian Dietary Guidelines provide a daily recommendation of discretionary foods for taller or more active people of up to 2.5 serves in women and 3 serves in men. However, guidelines recommend these foods “should be limited to small amounts and only eaten sometimes”. Therefore, maximum points were given to the lowest intakes with proportionate scores for intakes above that; ^6^ The Australian Dietary Guidelines do not provide recommendations for daily servings of added sugar. The American Heart Association recommends limiting the amount of added sugars to half the daily discretionary allowance (i.e., men = 1.5 serves and women = 1.25 serves) or six teaspoons per day for women and nine teaspoons per day for men [52]. Abbreviations: y = years, M = male, F = female.

**Table 2:** Characteristics of RESIDential Environments (RESIDE) study participants at T4 (*n* = 555) ^1^.

Characteristic	(*n*) % or Mean ± SD		
Sociodemographic			
Male	(212) 38.2		
Age (years)	(555) 47.9 ± 11.9		
Marital status			
Married/de facto	(476) 85.8		
Separated/divorced/widowed	(55) 9.9		
Single	(20) 3.6		
Education			
Secondary or less	(187) 33.7		
Trade/apprentice/certificate	(193) 34.8		
Bachelor or higher	(161) 29.0		
Income (AU$)			
<50,000	(83) 15.0		
50,000–69,999	(50) 9.0		
70,000–89,999	(77) 13.9		
≥90,000	(315) 56.8		
Health and lifestyle			
Self-rated health			
Excellent	(79) 14.2		
Very good	(205) 36.9		
Good/fair	(260) 46.8		
Poor	(7) 1.3		
Smoking status			
Never smoked	(288) 51.9		
Ex-smoker	(229) 41.3		
Current smoker	(38) 6.8		
BMI (kg/m^2^)			
Healthy (BMI ≥ 18.5 to <25)	(207) 37.3		
Overweight (BMI ≥ 25 to <30)	(199) 35.9		
Obese (≥30)	(107) 19.3		
Other (no response or BMI < 18.5)	(42) 7.6		
Physical activity ^2^			
Low	(213) 28.4		
Moderate	(266) 47.9		
High	(76) 13.7		
**ADG Component**	**Mean (SD)**	**Median (IQR)**	**% Achieving Maximum Criteria ^3^**
Vegetables	5.7 (2.2)	6.0 (4.0–7.5)	7.2
Fruit	7.6 (2.7)	10.0 (5.0–10.0)	53.5
Grains/cereals	7.4 (1.7)	8.0 (6.5–8.5)	4.7
Lean meats	6.4 (2.2)	6.3 (5.0–8.8)	5.9
Dairy or alternatives	5.3 (1.7)	5.5 (4.3–6.5)	0.4
Water/fluids	7.9 (1.9)	7.5 (7.5–10.0)	38.4
Saturated fat	7.9 (1.4)	8.0 (7.0–9.0)	10.6
Added salt	6.9 (3.1)	7.5 (5.0–10.0)	40.2
Added sugar	6.9 (1.8)	7.5 (6.3–7.5)	10.1
Alcohol	7.8 (2.5)	7.5 (5.0–10.0)	46.7
RDGI score	69.9 (8.8)	70.0 (64.5–76.0)	0

^1^ Categories of participant characteristics may not add up to 100% due to some missing data; ^2^ Categories based on standardized scoring [35,36]; ^3^ Equivalent to a maximum score of 10 for individual components or an RDGI score of 100. Abbreviations: IQR = interquartile range.

**Table 3:** Estimated coefficients from the fitted linear regression models for prediction of S-RDGI scores (S-RDGI1 and S-RDGI2) ^1^ from the scores of the sub-sets of survey items (*n* = 555).

Survey Item	Standard Deviation (SD)	Coefficient	*p*-Value	95% CI
Six item score: S-RDGI1				
Intercept		37.694		35.39, 39.99
Vegetables	2.191	1.387	<0.001	1.17, 1.61
Fruit	2.692	1.375	<0.001	1.20, 1,55
Red meat	1.546	1.438	<0.001	1.13, 1.75
Chips	0.457	2.931	<0.001	1.85, 4.01
Meat products	0.508	1.130	0.026	0.14, 2.12
Milk type	0.735	2.401	<0.001	1.77, 3.03
Nine item score: S-RDGI2				
Intercept		30.555		28.37, 32.74
Vegetables	2.191	1.287	<0.001	1.10, 1.47
Fruit	2.692	1.265	<0.001	1.11, 1.42
Red meat	1.546	1.322	<0.001	1.06, 1.58
Chips	0.457	2.172	<0.001	1.24, 3.10
Meat products	0.508	0.913	0.033	0.07, 1.75
Milk type	0.735	2.233	<0.001	1.70, 2.77
Fish	1.432	0.984	<0.001	0.70, 1.26
Alcohol (days/week)	1.722	1.246	<0.001	1.01, 1.48
Alcohol (drinks/day)	1.451	0.807	<0.001	0.52, 1.09

^1^ Prediction of S-RDGI1 scores was based on the six survey items (independent variables) and RDGI scores (dependent variable). Prediction of S-RDGI2 scores was based on the nine survey items (independent variables) and RDGI scores (dependent variable).

**Table 4:** Correlations between diet quality scores and intakes of food and drink items (based on original frequency categories outlined in Table A1) and Australian Dietary Guidelines (ADG) components (based on scoring outlined in Table 1).

Questionnaire Item (Frequency Categories)	S-RDGI1	S-RDGI2	RDGI
Vegetables ^1^	0.56 **	0.50 **	0.42 **
Fruit ^1^	0.71 **	0.65 **	0.56 **
Bread ^2^	−0.01	0.00	0.05
Pasta, rice, noodles or other cooked cereals ^2^	−0.12 **	−0.08	0.07
Red meat ^2^	−0.40 **	−0.36 **	−0.29 **
Fish ^2^	0.24 **	0.37 **	0.30 **
Milk ^3^	−0.01	0.03	0.09 *
Cheese ^2^	−0.08	−0.06	0.08
Meat products ^2^	−0.39 **	−0.37 **	−0.31 **
Chips, French fries, wedges, fried potatoes or crisps ^2^	−0.45 **	−0.41 **	−0.36 **
Fried roast or BBQ chicken, pizza, burgers or fish and chips ^2^	−0.32 **	−0.29 **	−0.36 **
Meat pies, sausage rolls or other savory pastries ^2^	−0.37 **	−0.35 **	−0.37 **
Biscuits, cakes, desserts, pastries, lollies and/or chocolate ^2^	−0.15 **	−0.08 *	−0.16 **
Sugary drinks ^3^	−0.17 **	−0.18 **	−0.21 **
Alcohol drinks per day ^4^	−0.18 **	−0.38 **	−0.31 **
**RDGI ADG Components**			
Vegetables	0.59 **	0.56 **	0.47 **
Fruit	0.73 **	0.67 **	0.59 **
Grains/cereals	0.04	0.04	0.23 **
Lean meats	0.42 **	0.47 **	0.38 **
Dairy or alternatives	0.20 **	0.21 **	0.29 **
Water/fluids	0.27 **	0.27 **	0.44 **
Saturated fat	0.51 **	0.47 **	0.47 **
Added salt	0.12 **	0.10 *	0.44 **
Added sugar	0.18 **	0.13 **	0.22 **
Alcohol	0.11 *	0.41 **	0.34 **

* *p* < 0.05, ** *p* < 0.01 (2-tailed); ^1^ Intake categories: do not eat = 1, 1 serve or less = 2, 2 serves = 3, 3–4 serves = 4, 5 serves = 5, 6 serves or more = 6; ^2^ Intake categories: never = 1, less than once per month = 2, once per month = 3, 2–3 times per month = 4, 1–2 times per week = 5, 3–5 times per week = 6, most days (6–7 times per week) = 7; ^3^ Number of cups; ^4^ Number of standard drinks.

**Table 5:** Association between mean diet quality scores from the RDGI, S-RDGI1, and S-RDGI2 by selected participant characteristics.

Participant Characteristic	*n*	RDGI Mean (95% CI)	*p*-Value	S-RDGI1 Mean (95% CI)	*p*-Value	S-RDGI2 Mean (95% CI)	*p*-Value
Gender							
Male	212	66.4 (65.23, 67.59)	<0.001	67.8 (66.91, 68.71)	<0.001	67.0 (66.01, 67.97)	<0.001
Female	343	72.0 (71.12, 72.86)		71.1 (70.40, 71.84)		71.6 (70.87, 72.38)	
Age (years)							
<42	202	69.2 (67.99, 70.52)	0.445	68.1 (67.20, 69.13) ^1,3^	<0.001	68.2 (67.14, 69.234) ^1,3^	<0.001
42–53	177	70.0 (68.75, 71.29)		69.9 (68.93, 70.89) ^1^		70.0 (68.95, 71.11)	
>53	176	70.4 (69.08, 71.68)		71.7 (70.74, 72.74)		71.6 (70.50, 72.69)	
Education							
Secondary or less	187	69.3 (68.12, 70.49)	0.051	69.7 (68.76, 70.61)	0.778	69.6 (68.58, 70.62)	0.277
Trade/apprentice/certificate	193	69.4 (68.04, 70.70)		69.8 (68.75, 70.81)		69.5 (68.38, 70.62)	
Bachelor or higher	161	71.2 (70.02, 72.66)		70.2 (69.08, 71.29)		70.7 (69.52, 71.82)	
Income (AU$)							
<50,000	83	70.6 (69.07, 72.21)	0.374	71.2 (69.86, 72.60)	0.055	71.3 (69.94, 72.755)	0.057
50,000–69,999	50	70.4 (67.93, 72.96)		68.8 (66.88, 70.72)		69.3 (67.52, 71.18)	
70,000–89,999	77	70.7 (68.73, 72.64)		70.6 (69.04, 72.27)		70.7 (69.03, 72.41)	
≥90,000	315	69.8 (68.20, 70.25)		69.3 (68.48, 70.04)		69.1 (68.24, 69.98)	
Self-rated health							
Excellent	79	72.1 (70.03, 74.23) ^1,2^	0.004	72.2 (70.64, 73.69) ^1,2^	0.001	71.7 (70.01. 73.44) ^1^	0.031
Very good	205	70.3 (69.10, 71.54)		70.1 (69.08, 71.03)		70.0 (68.96, 71.08)	
Good/fair	260	68.9 (67.93, 69.95)		69.1 (68.26, 69.88)		69.2 (68.32, 70.10)	
Poor	7	62.7 (50.95, 74.55)		65.0 (59.37, 70.67)		65.8 (58.88, 72.81)	
Smoking status							
Never smoked	288	71.59 (70.61, 72.59) ^1,3^	<0.001	70.58 (69.73, 71.43) ^1^	0.001	71.18 (70.30, 72.06) ^1,3^	<0.001
Ex-smoker	229	68.70 (67.60, 69.79) ^1^		69.45 (68.62, 70.28) ^1^		68.88 (67.96, 69.80) ^1^	
Current smoker	38	63.64 (60.55, 66.72)		66.41 (64.03, 68.78)		65.72 (62.99, 68.47)	
Physical activity ^4^							
Low	213	68.34 (67.12, 69.55) ^1,3^	0.001	68.27 (67.37, 69.17) ^1,3^	0.000	68.57 (67.56, 69.57) ^1,3^	0.002
Moderate	266	70.32 (69.31, 71.33)		70.59 (69.78, 71.41)		70.33 (69.43, 71.22)	
High	76	72.49 (70.40, 74.58)		71.72 (70.04, 73.40)		71.81 (70.09,73.54)	
BMI (kg/m^2^)							
Healthy (BMI ≥ 18.5 to <25)	207	70.48 (69.28, 71.68)	0.546	70.41 (69.45, 71.36)	0.241	70.52 (69.52, 71.51)	0.400
Overweight (BMI ≥ 25 to <30)	199	69.70 (68.47, 70.93)		69.93 (68.97, 70.88)		69.67 (68.58, 70.76)	
Obese (BMI ≥30)	107	68.99 (67.34, 70.64)		69.22 (67.89, 70.55)		69.24 (67.82, 70.66)	
Other (no response or BMI < 18.5)	42	69.73 (66.70, 72.75)		68.40 (66.27, 70.54)		69.02 (66.70, 71.34)	

^1^ Significantly different from 3; ^2^ Significantly different from 4; ^3^ Significantly different from 2; ^4^ Categories based on standardized scoring [35,36].

## Appendix A

**Table A1:** Semi-quantitative food frequency and dietary behavior questions in the RESIDE survey.

Survey Item	Description	ICC ^1^	Frequency Categories	RESIDE Time Point ^2^
**Food Frequency Questions (FFQ)**				
1 (FFQ)	How many serves of vegetables do you usually eat each day (including fresh, frozen and tinned)? [37]	-	- Do not eat - 1 serve or less - 2 serves - 3–4 serves - 5 serves - 6 serves or more	T1, T2, T3, T4
2 (FFQ)	How many serves of fruit do you usually eat each day (including fresh, dried, frozen and tinned fruit)? [37]	-		
3 (FFQ)	How often do you eat red meat (beef, lamb, and kidney but not pork or ham)? Include all minimally processed forms of red meat such as chops, steaks, roasts, rissoles, mince, stir-fries, and casseroles. [39]	0.95	- Never - Less than once per month - Once per month - 2–3 times per month - 1–2 times per week - 3–5 times per week - Most days (6–7 times per week)	T1, T2, T3, T4
4 (FFQ)	How often do you eat chips, French fries, wedges, fried potatoes or crisps? [39]	0.90		
5 (FFQ)	How often do you eat meat products such as sausages, frankfurters, polony, meat pies, bacon or ham? [39]			
6 (FFQ)	How often do you eat fish?	0.86	- Never - Less than once per month - Once per month - 2–3 times per month - 1–2 times per week - 3–5 times per week - Most days (6–7 times per week)	T2, T3, T4
7 (FFQ)	How often do you eat bread (including bread rolls, flat breads, crumpets, bagels, English or bread type muffins)? [39]	0.87	- Never - Less than once per month - Once per month - 2–3 times per month - 1–2 times per week - 3–5 times per week - Most days (6–7 times per week)	T4
8 (FFQ)	How often do you eat pasta, rice, noodles or other cooked cereals? [39]	0.82		
9 (FFQ)	How often do you eat cheese (including ricotta, cottage, processed, cream cheese, hard and soft cheese)? [39]	0.89		
10 (FFQ)	How often do you eat fried, roast or BBQ chicken, pizza, burgers or fish and chips? [38]	-		
11 (FFQ)	How often do you eat meat pies, sausage rolls or other savory pastries? [39]	0.88		
12 (FFQ)	How often do you eat biscuits, cakes, desserts, pastries, lollies and/or chocolate? [38]	0.87		
**Dietary Behavior Questions (DBQ)**				
1 (DBQ)	What type of milk do you usually consume? [38]	-	- Skim - Low/reduced fat (smart milk, Hi Lo, Pura Light Start, Tone) - Other (e.g., soy) - Whole (full cream) - Don’t know	T1, T2, T3, T4
2 (DBQ)	On how many days of the week do you usually drink alcohol?	-	- I don’t drink alcohol - Less than once per week - Number of days per week	T2, T3, T4
3 (DBQ)	On a day when you drink alcohol, how many standard drinks do you usually have?	-	Number of standard drinks	T2, T3, T4
4 (DBQ)	What type of bread do you usually eat? [40]	0.94	- Multigrain bread - Whole meal bread - Rye bread - High fiber white bread - White bread - I don’t eat bread - Other	T4
5 (DBQ)	About how much milk (in total) do you usually have in a day? [39]	0.79	- Less than 150 mL - 150 to 300 mL - 301 to 600 mL - More than 600 mL	T4
6 (DBQ)	How many cups of water, including sparkling water, do you drink in a day?	-	Number of cups	T4
7 (DBQ)	How many cups of regular or sugar sweetened soft drinks, cordial, fruit juice or sports drinks do you drink in a day? [11]	0.85		
8 (DBQ)	How many cups of diet or sugar-free soft drinks, cordial or sports drinks do you drink in a day (such as coke zero or sugar free Gatorade)?	-		
9 (DBQ)	How many cups of hot drinks do you drink in a day (such as tea, coffee, herbal tea)?	-		
10 (DBD)	How often is the meat you eat trimmed of fat either before or after cooking? [35]	0.93	- Don’t eat meat - Never - Rarely - Sometimes - Usually - Don’t know	T4
11 (DBQ)	How often do you add salt to your food after it is cooked? [35]	0.85	- Never - Rarely - Sometimes - Usually - Don’t know	T4
12 (DBQ)	How often is salt added to your food during cooking? [35]	0.89		

^1^ ICC = intraclass correlation based on a one-week test-retest reliability of items; ^2^ T1 prior to moving (baseline from 2003–2005), T2 (1-year post move from 2004–2006), T3 (2–3 years post from 2006–2008), and T4 (6–9 years post move from 2011–2012).

**Table A2:** Relevant Australian Dietary Guidelines [4] and Australian Guidelines to Reduce Health Risks from Drinking Alcohol [47] used to construct the RDGI.

**Australian Dietary Guidelines**
Guideline 2: Enjoy a wide variety of nutritious foods from these five groups every day
Plenty of vegetables of different types and colors, and legumes/beans.
Fruit.
Grain (cereal) foods, mostly wholegrain and/or high cereal fiber varieties, such as breads, cereals, rice, pasta, noodles, polenta, couscous, oats, quinoa and barley.
Lean meats and poultry, fish, eggs, tofu, nuts and seeds, and legumes/beans.
Milk, yoghurt, cheese and/or their alternatives, mostly reduced fat.
Drink plenty of water.
Guideline 3: Limit intake of foods containing saturated fat, added salt, added sugars and alcohol
Limit intake of foods high in saturated fat such as many biscuits, cakes, pastries, pies, processed meats, commercial burgers, pizza, fried foods, potato chips, crisps and other savory snacks.
Limit intake of foods and drinks containing added salt.
Limit intake of foods and drinks containing added sugars such as confectionary, sugar-sweetened soft drinks and cordials, fruit drinks, vitamin waters, energy and sports drinks.
If you choose to drink alcohol, limit intake. For women who are pregnant, planning a pregnancy or breastfeeding, not drinking alcohol is the safest option.
**Australian Guidelines to Reduce Health Risks from Drinking Alcohol**
Guideline 1: Reducing the risk of alcohol-related harm over a lifetime
No more than 2 standard drinks on any day.
Guideline 2: Reducing the risk of injury on a single occasion of drinking
No more than 4 standard drinks on a single occasion.
